# Prediction of Sentinel Lymph Node Metastasis in Breast Ductal Carcinoma *In Situ* Diagnosed by Preoperative Core Needle Biopsy

**DOI:** 10.3389/fonc.2020.590686

**Published:** 2020-11-10

**Authors:** Kai Zhang, Lang Qian, Qian Zhu, Cai Chang

**Affiliations:** Department of Medical Ultrasound, Fudan University Shanghai Cancer Center, Shanghai, China

**Keywords:** breast carcinoma, ductal carcinoma *in situ*, core needle biopsy, sentinel lymph node, axillary surgery

## Abstract

**Purpose:**

The positivity of sentinel lymph node (SLN) metastasis is relatively low in ductal carcinoma *in situ* (DCIS) patients. The aim of this study was to investigate factors associated with SLN metastasis and build a model to predict the potential risk of SLN metastasis in patients with a preoperative diagnosis of DCIS.

**Patients and Methods:**

Core needle biopsy-proved DCIS patients who underwent SLN biopsy and breast surgery were retrospectively reviewed and selected. Univariate analysis was used to identify the variables correlated with SLN metastasis. A model to predict SLN metastasis was developed using a multivariate logistic regression in the training set and then validated in an internal set.

**Results:**

A total of 407 patients with a preoperative diagnosis of DCIS were included. Upstaging to invasive/microinvasive cancer occurred in 225 patients after surgery. SLN metastasis was found in 42 patients, including 32 patients upstaging to invasive disease, 8 to microinvasive disease, and 2 pure DCIS. Tumor size based on US examination, axillary ultrasound finding, multifocality, surgery, upstaging, and Ki-67 expression were significantly related to SLN metastasis. The model incorporating tumor size, axillary ultrasound finding and multifocality yielded an AUC of 0.805 (95% CI: 0.715–0.895, *p*<0.001) in the training set, and 0.729 (95% CI: 0.547–0.911, *p*=0.013) in the testing set.

**Conclusion:**

A simple model was developed to predict SLN metastasis in patients with a preoperative diagnosis of DCIS. With good discriminatory power, this model should be helpful for surgeons to decide if SLN biopsy could be safely avoided in certain patients.

## Introduction

Breast cancer is the most frequently diagnosed cancer among females in China as in most other countries (1, 2). Ductal carcinoma *in situ* (DCIS) is a non-invasive breast cancer which represents approximately 20-30% of all new breast cancer diagnoses (3, 4). Unlike invasive disease, DCIS is confined in the milk ducts and, theoretically, lacks the ability of metastasis. Therefore, sentinel lymph node (SLN) biopsy is just recommended for patients with preoperatively diagnosed pure DCIS undergoing mastectomy, in case when invasive disease is found in the surgical specimen, a second SLN procedure becomes mandatory but is no more possible (5). However, in clinical practice, a substantial proportion of patients with a preoperative diagnosis of DCIS still undergo SLN biopsy during breast conserving surgery (6–10). This discrepancy between guidelines and clinical practice may be largely attributed to the reluctance to return to the operating room when occult invasive disease is identified in the surgical specimen.

Notably, the proportion of SLN metastasis in DCIS patients undergoing SLN biopsy during breast surgery (breast conserving surgery or mastectomy) is reported to be 0.6-13.4% (6, 8, 11–17). Given the low positivity and the potential harms the SLN procedure may cause, the role of SLN biopsy in most DCIS patients has been controversial in recent years (18). Consequently, it is of clinical significance to identify DICS patients with high potential of axillary lymph node metastasis who will be benefited from the SLN procedure. However, little progress has been made until now (19). Therefore, the aim of this study was to investigate factors associated with SLN metastasis and build a model to predict the potential risk of SLN metastasis in patients with a preoperative diagnosis of DCIS.

## Patients and Methods

### Patients

Patients with DCIS diagnosed by ultrasound-guided core needle biopsy (CNB) between January 2017 and December 2018 in our hospital were included in this retrospective study. The inclusion criteria included: 1) first diagnosed as breast cancer, 2) undergoing SLN biopsy and breast conserving surgery or mastectomy, 3) without therapy before surgery. The data of ultrasound examination, mammography and pathological evaluation were collected from the medical records. The procedure and interpretation of the results of SLN biopsy and ultrasound assessment were illustrated in a prior study (20). Briefly, the largest metastases in the SLN with a maximal diameter >2 mm, 0.2–2 mm, and ≤0.2 mm were classified as macrometastases, micrometastases, and isolated tumor cell, respectively. The axillary ultrasound was considered positive when an abnormal node with at least one of the following suspicious findings was recorded: diffuse cortical thickening of ≥3 mm; focal cortical bulge; eccentric cortical thickening; rounded hypoechoic node; complete or partial effacement of the fatty hilum; nonhilar cortical blood flow on color Doppler images; complete or partial replacement of the node with an ill-defined or irregular mass; or microcalcifications in the node. Otherwise, it was considered negative. Multifocality of primary tumor was determined on the basis of ultrasound examination, with two or more lesions that completely separate from each other considering as multifocal.

### Pathological Assessment

The expression of estrogen receptor (ER), progesterone receptor (PR) and Ki-67 was measured by immunohistochemical staining in the experimental specimens. Any staining of 1% of tumor cells or more was considered positive for both ER and PR. The case with staining of 14% or less tumor cells was considered low expression for Ki-67. The measurement of HER2 in the experimental specimens was determined according to the ASCO/CAP guidelines (21).

### Statistical Analysis

Quantitative data were presented as median and range, while qualitative data as frequency and percentage. Univariate analysis was used to identify the variables correlated with SLN metastasis. Mann–Whitney *U*-test was used for the analysis of quantitative data, while the Chi-squared test for qualitative data. Patients were then randomly divided into a training set and a testing set with a ratio of 7:3. A model to predict SLN metastasis was developed using a multivariate logistic regression backward stepwise method in the training set. All variables determined preoperatively with *p*<0.2 in the univariate analysis were put in the logistic regression analysis. Variables with *p*<0.05 in the analysis were included in the final predictive model, which was validated using the testing set. Area under the receiver operating characteristic curves (AUCs) were calculated to quantify the ability of the model to rank patients according to risk. All tests were two-sided, and *p*<0.05 indicated statistical significance. The SPSS version 19.0 (IBM Corp., Armonk, NY, USA) software was used for all statistical analysis.

## Results

### Patients Characteristics

A total of 407 female patients with median age of 49 years (range 22–81 years) were eligible for this study (**Table 1**). Most of patients (84.5%, 344/407) underwent mastectomy. Upstaging occurred in 225 (55.3%) patients after surgery. Of them, invasive disease was identified in 103 patients, and 122 cases were accompanied with microinvasive disease. SLN metastasis was found in 42 (10.3%) patients, among which macrometastasis alone and micrometastasis alone both presented in 20 patients, respectively. The other two patients had both macrometastsis and micrometastasis in the SLNs. Thirty-two of 103 (31.1%) patients upstaging to invasive cancer had SLN metastasis including 18 cases of macrometastsis alone, 12 cases of micrometastasis alone and two cases of both macrometastsis and micrometastasis. Eight of 122 (6.7%) patients upstaging to microinvasive cancer had SLN metastasis (two cases of macrometastsis alone and six cases of micrometastasis alone). There were only two patients (1.1%, 2/182) who had SLN metastasis without upstaging after surgery, both of whom were micrometastasis alone. This study was approved by the Ethics Committee of Fudan University Shanghai Cancer Center. Informed consent was waived due to its retrospective design and no identifiable information was disclosed.

**Table 1:** Patients’ characteristics and univariate analysis of variables for sentinel lymph node metastasis.

Variables		Number	SLN metastasis (%)		*p*
			No	Yes	
Age, years	Median(range)	49 (22–81)	49 (22–81)	47 (26–67)	0.347
History	No	376	336 (89.4)	40 (10.6)	0.668
	Yes	31	29 (93.5)	2 (6.5)	
Menopause	No	239	211 (88.3)	28 (11.7)	0.269
	Yes	168	154 (91.7)	14 (8.3)	
Birth	No	19	15 (78.9)	4 (21.1)	0.234
	Yes	388	350 (90.2)	38 (9.8)	
Palpability	No	38	37 (97.4)	1 (2.6)	0.175
	Yes	368	328 (88.9)	41 (11.1)	
Mammography finding	Calcifications only	90	81 (90.0)	9 (10.0)	0.947
	Calcifications and mass/distortion	114	101 (88.6)	13 (11.4)	
	Mass or distortion	63	56 (88.9)	7 (11.1)	
	No report	140	127 (90.7)	13 (9.3)	
Multifocality	No	374	341 (91.2)	33 (8.8)	0.002
	Yes	33	24 (72.7)	9 (27.3)	
Axillary US	Negative	381	355 (93.2)	26 (6.8)	<0.001
	Positive	26	10 (38.5)	16 (61.5)	
Size, mm	Median(range)	25.0 (6–79)	25.0 (6–79)	29.5 (10–70)	0.002
ER^a^	Negative	197	179 (90.9)	18 (9.1)	0.428
	Positive	208	184 (88.5)	24 (11.5)	
PR^a^	Negative	223	201 (90.1)	22 (9.9)	0.712
	Positive	182	162 (89.0)	20 (11.0)	
HER2^a^	Negative	136	122 (89.7)	14 (10.3)	0.206
	Positive	229	202 (88.2)	27 (11.8)	
	Uncertain	40	39 (97.5)	1 (2.5)	
Ki-67^a^	Low	98	95 (96.9)	3 (3.1)	0.006
	High	307	268 (87.3)	39 (12.7)	
Grade	Low-moderate	178	156 (87.6)	22 (12.4)	0.435
	High	222	203 (91.4)	19 (8.6)	
	Unknown	7	6 (85.7)	1 (14.3)	
Surgery	BCS	63	62 (98.4)	1 (1.6)	0.013
	MST	344	303 (88.1)	41 (11.9)	
Upstaging	No	182	180 (98.9)	2 (1.1)	<0.001
	Yes	225	185 (82.2)	40 (17.8)	

^a^This data was missing in two patients.

BCS, breast conserving surgery; MST, mastectomy; SLN, sentinel lymph node; US, ultrasound.

### Factors Correlated With Sentinel Lymph Node Metastasis

Factors including age, family history, menopause status, birth history, palpability, mammography finding, nuclear grade, ER expression, PR expression, and HER2 expression were not correlated with SLN metastasis (**Table 1**). However, tumor size based on US examination, axillary ultrasound finding, multifocality, surgery, upstaging, and Ki-67 expression were significantly related to SLN metastasis (**Table 1**). Specifically, SLN metastasis was more likely to happen in patients with larger tumor size, positive axillary ultrasound, multifocal lesions. Patients who underwent mastectomy, upstaged or had cancer with higher Ki-67 expression had a higher possibility to have metastasis in SLNs.

### Model to Predict Sentinel Lymph Node Metastasis

Two hundred and eighty-seven patients were allocated in the training set, and 120 patients in the testing set. They were comparable in patients’ characteristics except that the training set had little higher proportion of patients with ER/PR positive than the testing set (**Table 2**). Based on the results of univariate analysis, preoperative variables including palpability, tumor size, axillary ultrasound finding and multifocality were put in the logistic regression analysis, which turned out that only the last three variables remained significant (**Table 3**). Thus, a model to predict SLN metastasis in DCIS patients diagnosed by CNB was developed with the three variables:

Logit(p)=−4.399+0.055  tumor size +3.009 axillary ultrasound+1.235 multifocality

**Table 2:** Comparison between training set and testing set.

Variables		Training set	Testing set	*p*
Total		287	120	–
Age, years	Median(range)	48 (22–81)	49 (26–74)	0.291
History	No	265	111	0.954
	Yes	22	9	
Menopause	No	174	65	0.227
	Yes	113	55	
Birth	No	16	3	0.180
	Yes	271	117	
Palpability	No	28	10	0.653
	Yes	259	110	
Mammography finding	Calcifications only	63	27	0.506
	Calcifications and mass/distortion	76	38	
	Mass or distortion	43	20	
	No report	105	35	
Multifocality	No	263	111	0.771
	Yes	24	9	
Axillary US	Negative	267	114	0.459
	Positive	20	6	
Size, mm	Median(range)	25 (7–79)	27 (6–68)	0.102
ER^a^	Negative	129	68	0.027
	Positive	157	51	
PR^a^	Negative	148	75	0.038
	Positive	138	44	
HER2^a^	Negative	100	36	0.579
	Positive	157	72	
	Uncertain	29	11	
Ki-67^a^	Low	75	23	0.140
	High	211	96	
Grade	Low-moderate	128	50	0.664
	High	155	67	
	Unknown	4	3	
Surgery	BCS	46	17	0.636
	MST	241	103	
Upstaging	No	134	48	0.216
	Yes	153	72	
SLN metastasis	No	256	109	0.621
	Yes	31	11	

^a^This data was missing in one patient of each set.

BCS, breast conserving surgery; MST, mastectomy; SLN, sentinel lymph node; US, ultrasound.

**Table 3:** Logistic regression analysis.

Variables	B	Wald	OR (95%CI)	P
Tumor size	0.055	0.015	1.056 (1.025–1.089)	<0.001
Axillary US	3.009	0.555	20.264 (6.824–60.175)	<0.001
Multifocality	1.235	0.618	3.440 (1.025–11.547)	0.046

**Figure 1:**
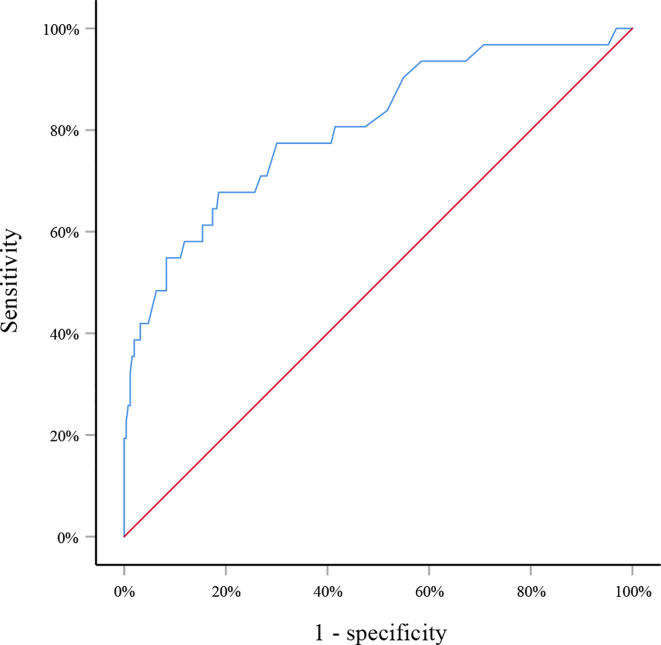
Receiver operating curve for the training set. The area under the curve was 0.805 (95% CI: 0.715–0.895, *p*<0.001).

**Figure 2:**
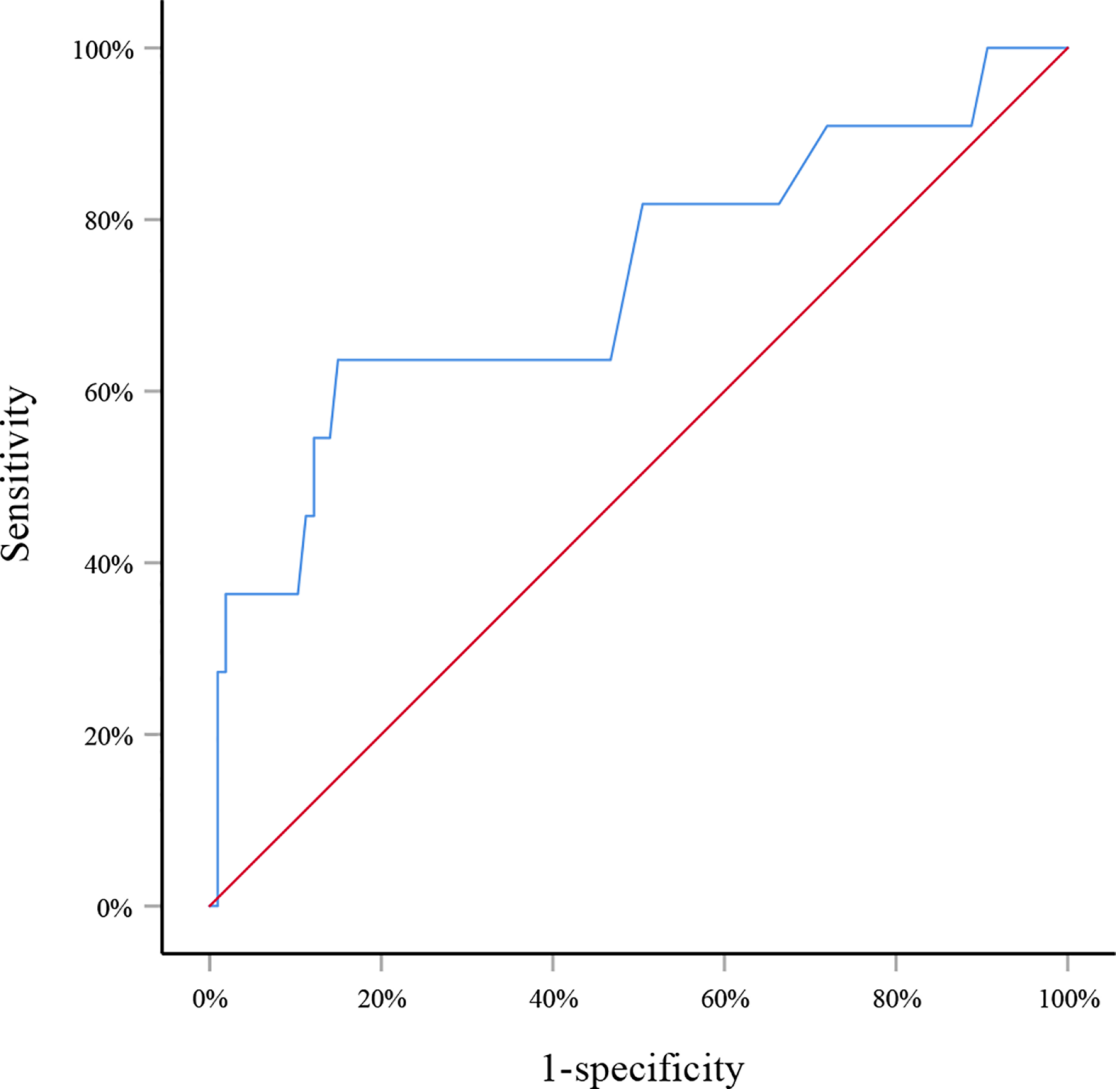
Receiver operating curve for the testing set. The area under the curve was 0.729 (95% CI: 0.547–0.911, *p*=0.013).

US, ultrasound.

in which *p* indicated the probability of SLN metastasis. The AUC of this model was 0.805 (95% CI: 0.715–0.895, *p*<0.001) (**Figure 1**). When applying to the testing set, this model yielded an AUC of 0.729 (95% CI: 0.547–0.911, *p*=0.013) (**Figure 2**).

## Discussion

The controversy on the role of SLN biopsy in patients preoperatively diagnosed as pure DCIS has been for a long time. The arguments for or against it, as introduced previously, mainly focus on the potential of upstaging to invasive cancer after surgery and low rate of SLN metastasis. However, what should not be neglected is that SLN metastasis could be found in some DCIS patients without upstaging after surgery, though in a very low rate (4%, 71/1787) (22). Also in this study, SLN metastasis was found in two patients with final pathology-proved pure DCIS (1.1%, 2/182). The mechanism of this phenomenon is beyond the scope of this study. But it does strengthen the clinical relevance of SLN biopsy in patients with a preoperative diagnosis of DCIS. And before the detrimental influence of axillary lymph node metastasis on the survival of patients can be certainly excluded, it is quite of clinical significance to perform SLN biopsy in highly selective patients with a preoperative diagnosis of DCIS because of low positivity of SLN metastasis.

SLN metastasis was found in 10.3% (42/407) of patients with preoperative DCIS in this study, a little higher than that reported over the latest five years (6, 8, 11, 12). This may be attributed to the relatively high total upstaging rate (55.3%, 225/407) of this study. About a quarter (25.5%, 103/407) of patients with invasive cancer were underestimated as DCIS in initial CNB, as well as another portion (30.0%, 122/407) with microinvasive cancer. These patients are more likely to have metastasis in SLNs, so the more they were included in the group, the higher the total rate of SLN metastasis was. And the main reason for relatively high upstaging rate in this study may be that in our center CNB specimens were stained to determine whether there were cancer cells but not mandatorily further stained to determine whether there was micoinvasiveness or invasiveness, which cannot be determined without further immunohistochemical staining.

Several prior studies have investigated the risk factors of SLN metastasis in patients with preoperative DCIS (12, 15, 17, 22). Factors such as age, tumor size, palpability, multifocality and upstaging have been proved to be correlated to SLN metastasis. In this study, we demonstrated that tumor size, multifocality and upstaging, but not age and palpability, were significantly related to SLN metastasis. In addition, we also found that positive axillary ultrasound finding, mastectomy and higher Ki-67 expression were significantly associated with more SLN metastasis.

However, what is most concerned by clinicians is to precisely predict SLN metastasis before surgery. So far, there have been plenty of models to predict axillary lymph node metastasis in breast cancer patients (23–25). Unfortunately, to the best of our knowledge no model has been developed to predict SLN metastasis in patients with a preoperative diagnosis of DCIS. Therefore, we built a model with factors that could be determined before surgery and were significantly correlated with SLN metastasis. This simple model incorporated factors including tumor size, axillary ultrasound finding and multifocality and yielded an AUC of 0.805 in the training set, suggesting good discrimination of SLN metastasis. The internal testing set was comparable with the training set in patients’ characteristics except for the ER/PR expression. Since they were determined after surgery and not correlated with SLN metastasis in the univariate analysis, no adjustment about ER/PR expression was applied in the logistic regression analysis. Internal validation of this model with the testing set achieved an AUC of 0.729, demonstrating relatively reliable discriminatory power of this model.

There were several limitations that should be noticed in this study. Firstly, this model did not incorporate any molecular information because it was not available before surgery in our hospital. In this study, Ki-67 expression was proved to be correlated with SLN metastasis in the univariate analysis. Therefore, the performance of this model may be improved if molecular information could be added. Secondly, this model was not validated externally. Thirdly, the retrospective nature of this study would inevitably lead to bias in the selection of patients.

## Conclusion

A simple model incorporating tumor size, axillary ultrasound finding and multifocality was developed to predict SLN metastasis in patients with a preoperative diagnosis of DCIS. With good discriminatory power, this model should be helpful for surgeons to decide if SLN biopsy could be safely avoided in certain patients.

## Data Availability Statement

The raw data supporting the conclusions of this article will be made available by the authors, without undue reservation.

## Ethics Statement

The studies involving human participants were reviewed and approved by the Ethics Committee of Fudan University Shanghai Cancer Center. The ethics committee waived the requirement of written informed consent for participation.

## Author Contributions

CC and KZ conceived and designed the study. KZ and LQ wrote the manuscript. All authors contributed to the article and approved the submitted version.

## Funding

This study was supported by the National Natural Science Foundation of China (Grant No. 81627804, 81830058).

## Conflict of Interest

The authors declare that the research was conducted in the absence of any commercial or financial relationships that could be construed as a potential conflict of interest.
